# Gastric Perforation: A Rare Presentation of Malignant Gastric Outlet Obstruction

**DOI:** 10.7759/cureus.22242

**Published:** 2022-02-15

**Authors:** Krixie Silangruz, Parthav Shah, Larissa Fujii-Lau, Sho Furuta

**Affiliations:** 1 Internal Medicine, University of Hawaii, Honolulu, USA; 2 Gastroenterology and Hepatology, The Queen's Medical Center, Honolulu, USA; 3 General Surgery and Trauma, The Queen's Medical Center, Honolulu, USA

**Keywords:** exploratory laparotomy, malignancy, pancreatic cancer, gastric perforation, gastric outlet obstruction

## Abstract

Mechanical gastric outlet obstruction (GOO) due to malignancy can occur with cancers in the stomach and duodenum. GOO can cause perforation of the stomach or the esophagus. We present a 65-year-old female with newly diagnosed metastatic pancreatic cancer who presented with acute onset abdominal pain. She was taken for emergent exploratory laparotomy where she was found to have gastric perforation and duodenal obstruction from an invasive large pancreatic head mass. This is a rare case report of gastric perforation due to a malignant gastric outlet obstruction secondary to invasive pancreatic cancer.

## Introduction

Gastric outlet obstruction (GOO) is either due to a benign or malignant mechanical obstruction or to a motility disturbance that interferes with gastric emptying. Malignant GOO can occur with cancers in the pylorobulbar area, the antropyloric zone, and the descending duodenum. Distal gastric cancer is the most common cause of GOO, followed by pancreatic adenocarcinoma with duodenal or gastric extension [1]. We present a rare case of gastric perforation secondary to gastric outlet obstruction from invasive pancreatic cancer. 

This case was presented as a poster in the American College of Gastroenterology (ACG) Annual Meeting 2021, Las Vegas, Nevada, USA.

## Case presentation

**Figure 1:**
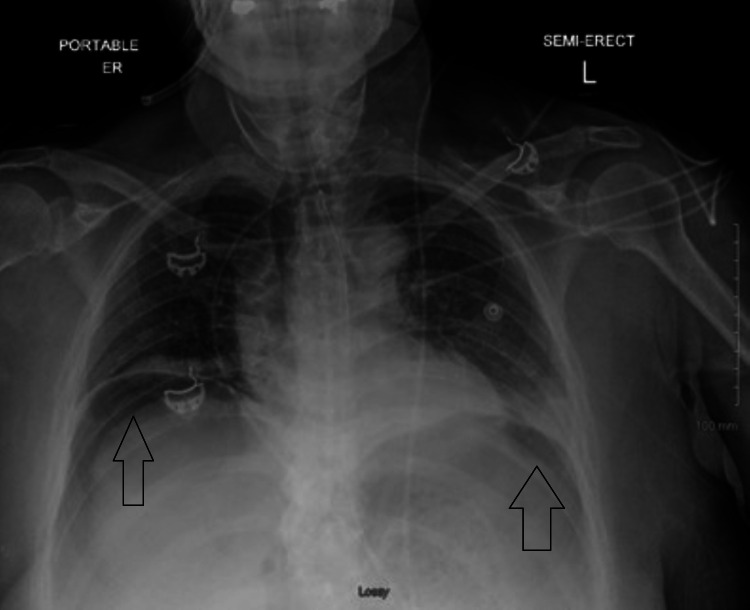
Free air under the hemidiaphragm.

**Figure 2:**
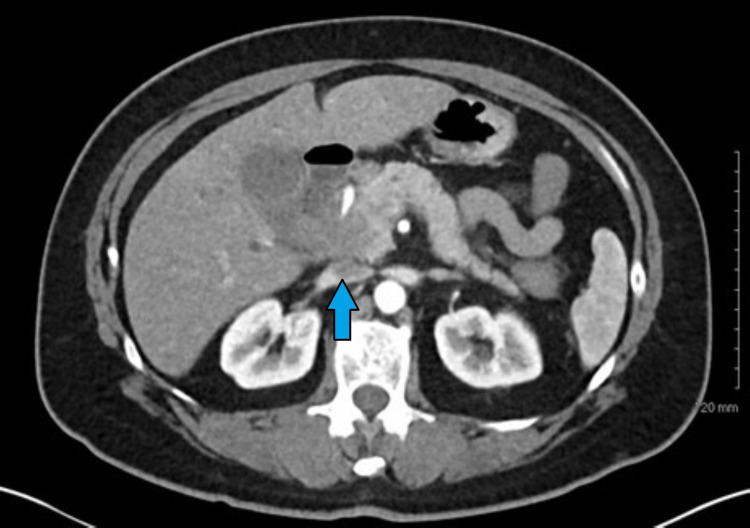
Ill-defined soft tissue enlargement is seen at the level of the pancreatic head, approximately 4 cm in diameter.

A 65-year-old female with newly diagnosed stage IV pancreatic cancer with metastases to the liver, presented with complaints of acute onset, severe diffuse abdominal pain. She was diagnosed with metastatic pancreatic cancer three months prior to her presentation and had elected to pursue hospice. She has had chronic abdominal pain relieved by pain medications. However, on the day of her admission, she started having diffuse abdominal pain that worsened on palpation and was associated with nausea and vomiting. She was diaphoretic, tachycardic, and hypotensive in the emergency room. On physical examination, she was noted to have a distended and rigid abdomen with involuntary guarding. Laboratory studies done on admission were significant for neutrophilic leukocytosis with left shift, high anion gap metabolic acidosis, and normal liver enzymes. A chest x-ray showed free air under the right hemidiaphragm (Figure 1). An abdominal x-ray showed gastric distension and gaseous distension of the colon. Computed tomography confirmed the large pneumoperitoneum with concern for bowel perforation (Figure 2).

**Figure 3:**
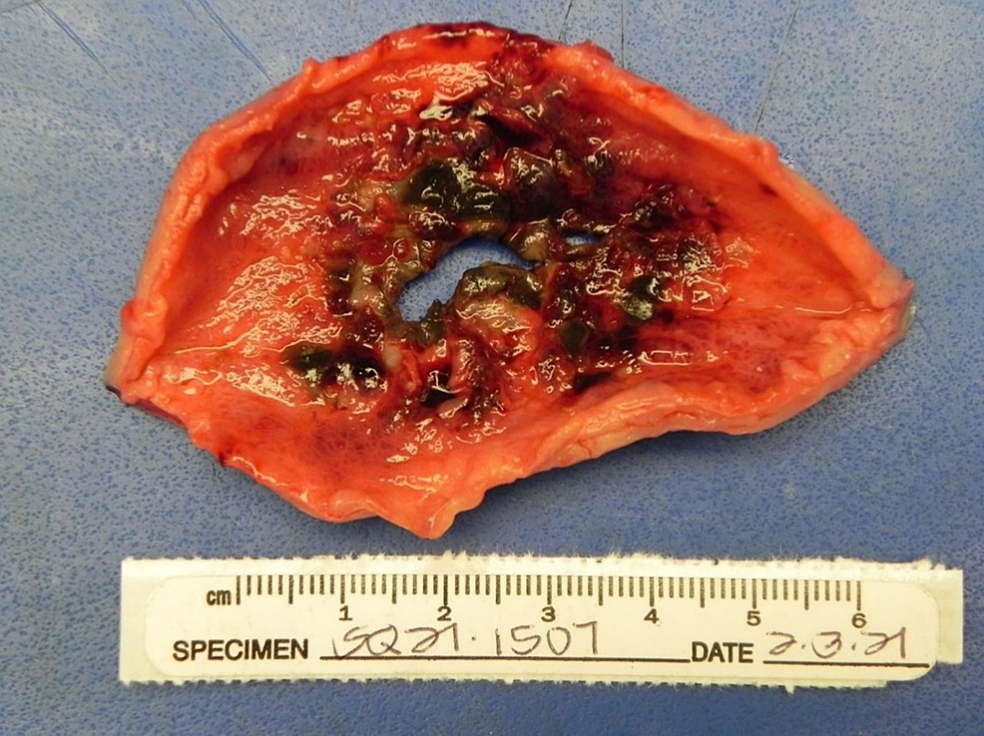
A large gastric perforation, approximately 5 cm in diameter, just anterior to the greater curvature, about halfway up the stomach, with necrotic and ischemic wound edges.

**Figure 4:**
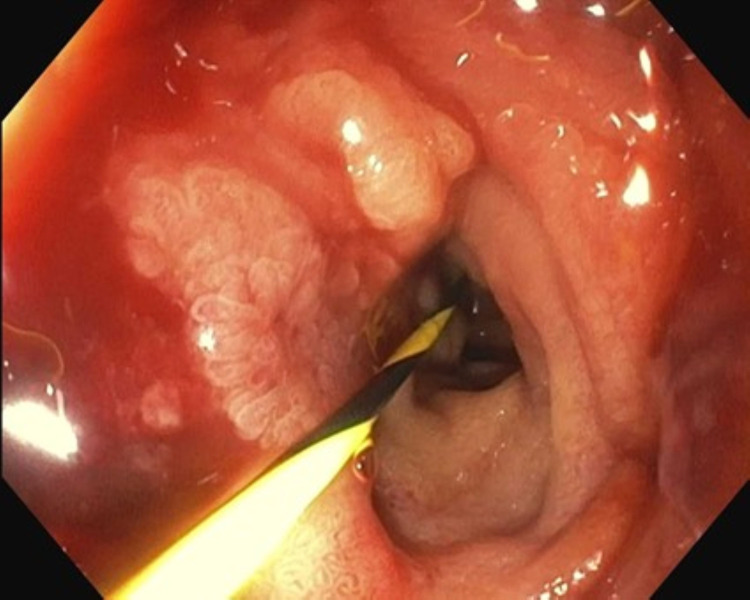
Duodenal bulb stenosis secondary to extrinsic compression, requiring dilation to allow for duodenoscope passage.

General surgery was consulted. She underwent emergent exploratory laparotomy which revealed a large volume of ascites of gastric contents with necrotic debris throughout the peritoneal cavity, approximately 5 cm of perforation just anterior to the greater curvature of the stomach with necrotic edges (Figure 3), and duodenal obstruction caused by an invasive large pancreatic head mass (Figure 4). There were no additional obstructions in the small bowel distal to the duodenum, and there were no appreciable defects in the small bowel and colon. A gastric wedge resection, abdominal washout, and Abthera wound vacuum placement were performed.

Pathology revealed foci of mucosal ischemia, ulceration, and acute inflammation with no malignancy identified. Forty-eight hours after her first surgery, she underwent a reopening of the laparotomy, abdominal washout, and Moss gastrojejunostomy tube insertion and gastrojejunostomy. Her hospital stay was complicated by *Candida tropicalis* and *Escherichia coli* peritonitis, which were treated with the appropriate antibiotic therapy. With the surgical interventions, antimicrobial therapy, and supportive care, including fluid resuscitation, the patient’s condition improved. She was discharged on day 16 of her hospital stay.

## Discussion

Gastric outlet obstruction is a clinical syndrome resulting from mechanical obstruction that interferes with normal gastric emptying. The etiology of GOO has changed dramatically over the past few decades. Until the late 1970s, benign disease was the most common culprit of GOO, when peptic ulcer disease accounted for the largest number of cases [2]. With the discovery and application of proton pump inhibitors (PPIs) and H2 blockers, this number has declined. Recent studies have shown that malignancy is the most common cause of GOO [3-5]. There is an estimate that 50 to 80% of all cases of GOO are attributable to malignancy [6,7]. Distal gastric cancer accounts for the most common cause of GOO, with up to 35% of cases, followed by pancreatic adenocarcinoma with duodenal or gastric extension, which accounts for 15% to 25% of cases. Proximal duodenal and ampulla neoplasms, gastric lymphoma, metastatic or primary duodenal malignancy, gastric carcinoid, and locally advanced gallbladder carcinoma or cholangiocarcinoma are less prevalent causes [1].

Malignant GOO can lead to perforation of the stomach or the esophagus. There have been case reports of esophageal perforation secondary to malignant GOO, and the location of all the tumors was found to be in the pre-pyloric region [8-10]. There have been case reports of gastric perforation due to gastric obstruction [11]. To date, there have been no published case reports of gastric perforation from a malignant gastric outlet obstruction secondary to an invasive pancreatic head cancer. The pathophysiology of the gastric perforation observed in our patient may be explained by obstruction of the duodenum by the pancreatic head cancer, leading to gastric dilation and subsequent perforation due to pressure effect.

Gastric perforation in adults usually presents with sudden onset abdominal or chest pain, which is believed to be due to leakage of gastric acid into the abdominal cavity. Because gastric pH is low along the gastric luminal surface, sudden discharge of acid into the abdomen causes acute peritoneal irritation, inflammation, and pain. Other presenting features include abdominal mass, emesis, nausea, hematemesis, and hematochezia. The patient in our case presented with acute abdominal pain, nausea, vomiting, and chills. She was also tachycardic, diaphoretic, hypotensive and had guarding and rigidity on abdominal examination, which are commonly seen in patients with perforation.

Gastric perforation may be suspected based upon history and physical examination findings, but a definite diagnosis relies upon imaging that demonstrates pneumoperitoneum or complications associated with perforation, such as an intra-abdominal or mediastinal abscess, or gastrointestinal fistula formation. An upright chest and abdomen radiograph is usually the initial imaging modality. It helps to detect free intraperitoneal air and is useful 50-70% of the time [12]. CT scan is more sensitive in recognizing free air, as was in the case of our patient, and can also help in localization of the exact site of the perforation [13].

Initial management of the patient with gastrointestinal perforation includes intravenous (IV) fluid therapy, cessation of oral intake, and broad-spectrum antibiotics. In our case, the patient was taken for an emergent exploratory laparotomy, which remains as the definitive treatment. It helps not only in treating but also in diagnosing the underlying anatomical cause of the perforation. Patients with evidence of perforation and with clinical signs of sepsis, peritonitis, bowel ischemia, or complete bowel obstruction require immediate surgery. 

GOO can cause perforation, so it is important to diagnose it early and treat it. There are some takeaways from our case. One is that the physicians should keep this in the differential when evaluating a patient with abdominal pain, especially in patients with a history of cancer. Another is that a careful history is important in evaluating patients with abdominal pain as it can help in diagnosing perforation early. Whenever perforation is confirmed or even strongly suspected, immediate surgical consultation is appropriate to determine if immediate surgical intervention is needed and the timing of surgery. Many patients will require urgent surgical intervention to limit ongoing abdominal contamination and manage the perforated site. It can also help in diagnosing the etiology of the perforation.

## Conclusions

Malignant GOO can lead to perforation of the stomach or the esophagus. A careful history is important in evaluating patients with abdominal pain suspected of having a gastrointestinal perforation. Patients with evidence of perforation with clinical signs of sepsis, peritonitis, bowel ischemia, or complete bowel obstruction require immediate surgery. This case highlights the importance of a thorough history in patients with abdominal pain, especially in the setting of known gastrointestinal cancer. This case shows the rare occurrence of gastric perforation from malignant GOO from invasive pancreatic carcinoma.
